# *Mycobacterium avium *subsp. *paratuberculosis *as a trigger of type-1 diabetes: destination Sardinia, or beyond?

**DOI:** 10.1186/1757-4749-2-1

**Published:** 2010-03-29

**Authors:** Pittu Sandhya Rani, Leonardo A Sechi, Niyaz Ahmed

**Affiliations:** 1 Pathogen Biology Laboratory, School of Life Sciences, University of Hyderabad, Hyderabad, India; 2 Department of Biomedical Sciences, University of Sassari, Sassari, Italy

## Abstract

Type 1 diabetes mellitus (T1DM) is a multifactorial autoimmune disease in which the insulin producing β cell population is destroyed by the infiltrated T lymphocytes. Even though the exact cause of T1DM is yet to be ascertained, varying degree of genetic susceptibility and environmental factors have been linked to the disease progress and outcome. *Mycobacterium avium *subsp. *paratuberculosis *(MAP) is an obligate zoonotic pathogen that causes chronic infection of intestines in ruminants, the Johne's disease. MAP that can even survive pasteurization and chlorination has also been implicated to cause similar type of enteritis in humans called Crohn's disease. With the increasing recognition of the link between MAP and Crohn's disease, it has been postulated that MAP is an occult antigen which besides Crohn's could as well be thought to trigger T1DM. Epitope homologies between mycobacterial proteins (Hsp 65) and pancreatic glutamic acid decarboxylase (GAD 65) and infant nutrition studies implicate MAP as one of the triggers for T1DM. PCR and ELISA analyses in diabetic patients from Sardinia suggest that MAP acts as a possible trigger for T1DM. Systematic mechanistic insights are needed to prove this link. Unfortunately, no easy animal model(s) or *in-vitro *systems are available to decipher the complex immunological network that is triggered in MAP infection leading to T1DM.

## Type 1 diabetes and MAP

**Figure 1:**
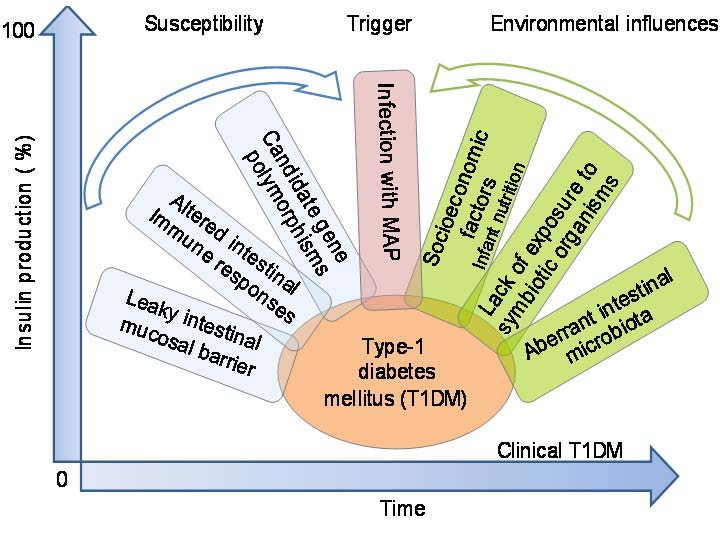
**Putative factors facilitating development and progression of T1DM**. Susceptibility factors such as altered intestinal immune responses with enhanced expression of antigen presenting HLA class II molecules and intracellular adhesion molecule (ICAM)-1 on the intestinal epithelium, leaky intestinal mucosal barrier with low levels of claudin and a background of environmental factors such as aberrant intestinal microbiota and lack of exposure to symbiotic organisms are hypothesized to be involved in the development of T-regulatory responses. And, absence of such response is implicated in the manifestation of autoimmune diseases. A complex interplay between these factors along with socio-economic factors (Sardinia) such as infant nutrition, milk infected with MAP- a putative trigger in genetically susceptible individuals, leads to the manifestation of clinical T1DM over a period of time with destruction of β cells of pancreas and decreased production of insulin. MAP has emerged central to this scenario lately due to the supporting studies from Sardinia that associates MAP with T1DM [17,28] but not with Type 2 diabetes (T2DM) [53].

**Figure 2:**
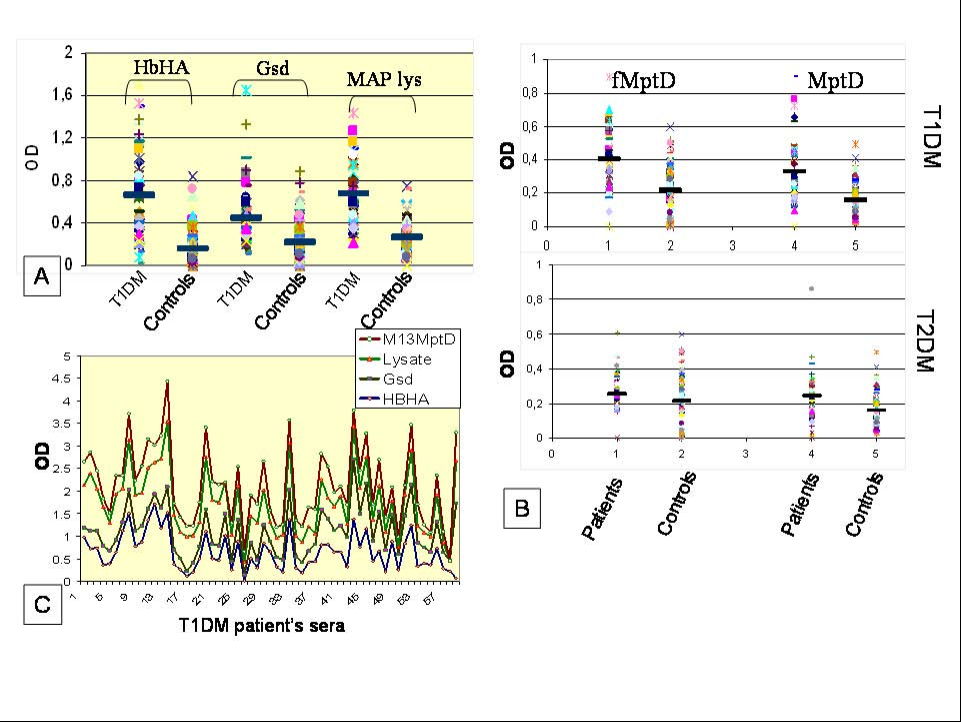
**Summary of the analyses by Sechi and colleagues involving Sardinian diabetic populations and healthy volunteers **[17,28,53]. Panels A and B: The data clearly revealed that T1DM patients show significant humoral immune responses to MAP (recombinant) proteins such as HbHa, Gsd & MptD; MAP lysate preparation; and specific phages corresponding to MAP specific protein MptD (mentioned as M13 MptD or fMptD) when compared to healthy controls and T2DM patients. Panel C: Multiplex ELISA analysis involving MptD specific phage (M13MptD), MAP lysate, Gsd and HbHa used against T1DM patients sera. Values on the Y- axis denote optical density values corresponding to anti-MAP serum immunoglobulin status. The dark, solid, horizontal lines represent median OD values for each group. Details of ELISA methods and choice of MAP antigens etc. have already been dealt with previously [17,28,53].

Type 1 diabetes accounting for about 5-10% of all diabetics is an end stage insulitis characterized by the presence of only 10-20% of insulin producing β-cells [1,2]. Also, T1DM is the second most common chronic disease during childhood and the most common form of diabetes affecting around 1.7 of every 1000 children [reviewed elsewhere, [3]]. Its worldwide prevalence is predicted to increase from 4.4 millions in 2000 to approximately 5.4 millions in 2010. The incidence of the disease is consistently increasing in many countries, the highest being in European countries, particularly in Finland, Sardinia and Sweden [4]. In a southern Indian urban population, the occurrence of T1DM in children less than 15 years of age was found to be 26 per 10000 [5]. Susceptibility to T1DM is inherited, but the mode of inheritance is not clearly known [6-8]. Genetic predisposition alone cannot explain the acquisition of T1DM. Several factors such as microbes, dietary factors and environmental toxins have also been implicated in the development of the disease. The American Diabetes Association (1997) classified T1DM into two forms: (a) type 1A (auto immune diabetes) which is characterized by the presence of auto reactive antibodies in the serum of the affected individuals. These antibodies are directed against Hsp60, insulin (IAA), insulinoma-associated protein-2 (IA-2) and glutamic acid decarboxylase (GAD65), an enzyme present in pancreatic β-cells. In patients with recent onset of T1DM auto reactive T- cells have also been detected. These auto-reactive T cells have been considered to be involved in β-cell destruction. [5,9-11]. (b) The other form, diabetes type 1B (diabetes with idiopathic loss of β-cell function) [9,10] is comparatively less common, lacks the autoimmunity connection and might need insulin replacement therapy in affected patients. In summary, the development of T1DM is mechanistically very complicated and several different factors have been proposed to influence the clinical outcome of the disease (Fig. 1). Lately, however, the role of microorganisms and pathogens is becoming evident in T1DM, although, without much (proof of principle) evidence; nevertheless, clinical level, supportive association studies based on diagnostics that specifically target MAP DNA and serum antibodies in the T1DM patients have been highly suggestive of a pathogen trigger (Fig. 2).

## Is MAP the trigger? - the Sardinian model

MAP, an obligate zoonotic pathogen is the causative agent of a chronic, inflammatory bowel disease referred to as Johne's disease of ruminants. The ability of MAP to exist in spheroplast form in addition to the bacillary forms adds to its survival potential [12]. The primary route of MAP infection is fecal- oral and once ingested, the bacterium lodges in to the mucosa associated lymphoid tissue (MALT) of the small intestine. It is then endocytosed by the M cells of Peyer's patches, which are further phagocytosed by intra epithelial macrophages. Activation of the macrophages causes an inflammatory immune response leading to clinical signs of Johne's disease. Inspite of its broad pathogenicity, MAP can live in animals for years without causing any clinical disease [13,14]. Wildlife reservoirs contribute to the cycle of re-infection [15]. Infected animals shed MAP bacilli in their milk and feces. MAP is found to be present in untreated water such as well water and in water bodies contaminated by agricultural runoff. The treatment of water to make it potable by the processes of sedimentation, filtration and chlorination has little or no effect on MAP [16]. Our group has shown in the past that MAP significantly associates with T1DM in Sardinian patients [17]. In the back-drop of sheep husbandry and high incidence of autoimmune diseases in Sardinia, it makes a lot of sense to brand MAP as a putative environmental trigger. It is however, not clear whether MAP also associates with T1DM (or other autoimmune diseases that occur) in other geographical regions. Nevertheless, the Sardinian model greatly helps us understand the inner side of socioeconomic and lifestyle factors in shaping a vulnerable, susceptible population despite the fact that sheep husbandry brought prosperity in terms of livelihood in this Mediterranean island. Modern animal breeding and expansion of dairy industries in the developed countries have led to increased exposure of the individuals to some of the animal pathogens. This is more prominently observed in Sardinia where sheep farming is intensively practiced and the sheep measure about 4-fold of the human population [17]. Further, studies have shown that Sardinia is highly endemic for the presence of MAP infection in sheep. MAP shedded in the milk of infected cows/sheep has been found to survive pasteurization [18]. There are several evidences indicating an association between early exposure to cow's milk and increased risk of T1DM [2,19,20]. Interestingly, it has also been observed that children at risk for T1DM who were breast fed exclusively for more than six months were less likely to have T1DM later in life than children at similar risk who were weaned on to cow's milk [21]. Such observations led to the foundation of the TRIGR study: Trial to Reduce Insulin Dependent Diabetes Mellitus in the Genetically at Risk. It is an ongoing study in 17 countries encompassing 6,200 infants who are genetically at risk to develop T1DM [22,23].

MAP, which causes Johne's disease in ruminants, is also implicated to cause a similar type of enteritis in humans called Crohn's disease [24,25]. Association of MAP with Crohn's disease indicates that there may be a putative link between MAP and T1DM [26]. As this hypothesis is getting ground, rapid and sensitive detection of active MAP infection in T1DM patients has become a challenge. Sardinia, a genetic isolate with alleles and haplotypes that are rare or absent elsewhere poses as a high endemic zone of autoimmune disorders such as T1DM, lupus erythematosus and multiple sclerosis. An interplay of genetic and environmental factors in Sardinia possibly could make it a hotspot to study autoimmune disorders. Sechi *et al*., (2008) have successfully demonstrated PCR based detection of IS 900, a specific signature locus of MAP in T1DM cases in Sardinia [27]. After identification of MAP in the blood of T1DM it was quite necessary to understand the host immune responses to MAP. This led them further to design immunoassays for the detection of anti-MAP antibodies in diabetic patients by ELISA, employing sensitive antigenic targets such as HbHa (heparin binding hemagglutinin) and Gsd (glycosyl transferase) proteins [17]. However, anti-MAP humoral responses corresponding to HbHa and Gsd could not be indicative of an active infection and also since these proteins are encoded by wider range of mycobacteria, this raised an issue of cross-reactivity with tubercle bacilli which could be an issue to deal with the BCG vaccinated individuals. This group later improvised the immunoassays by including a MAP specific protein, MptD into their battery of antigens. Apart from purified antigens, specific phages corresponding to MptD (fMptD) were also used in a sandwich ELISA scheme [28]. The improvised ELISA assays that use multiple target proteins and phages for the detection of anti-MAP antibodies revealed extremely significant humoral immune responses in T1DM patients when compared to T2DM and healthy controls [28] (Fig. 2). A final evidence was presented in terms of culture of MAP bacilli from the blood of two of the T1DM patients from Sardinia [28].

## Genetic susceptibility and microbial mechanisms of T1DM

The recent development of high throughput genotyping technologies and the availability of large sample sizes have become a favorable tool for association studies of human genes controlling complex traits [29]. Susceptibility genes for insulin dependent diabetes have been mapped to the chromosomal regions: HLA DRQ, INS VNTR and CTLA-4 (cytotoxic lymphocyte antigen-4) [30,31]. Additionally, SLC11A1 (NRAMP) and VDR (vitamin D receptor) genes have also been implicated to be involved in T1DM [32,33]. In humans, SLC11A1 - solute carrier 11A1gene (previously known as NRAMP1) is mapped on 2q35, is composed of 15 exons and it spans at least 16 kb of the DNA. It encodes an integral membrane protein of 550 amino acids that is expressed exclusively in the lysosomal compartment of monocytes and macrophages. This membrane protein functions as a cation transporter that regulates iron homeostasis [34,35]. In response to intracellular pathogens, SLC11A1 exhibits multiple pleiotropic effects such as gamma interferon induced class II expression and acidification of phagosomes [35,36]. Mutations of SLC11A1 impair phagosome acidification, creating thereby a suitable environment for the persistence of intracellular bacteria [37]. Sechi and colleagues have also proposed that SLC11A1 gene polymorphism is associated with MAP DNA presence and T1DM in Sardinia [38].

Infectious agents such as MAP by virtue of their antigens have been implicated to play an important role in the development of autoimmune diseases such as Crohn's disease and T1DM. Sequence similarity among the infectious agents and self proteins (molecular mimicry) has been proposed to be one of the central mechanisms underlying development of autoimmunity [39]. T1DM is characterized by elevated levels of immune responses targeted against several auto antigens including Hsp 60, insulin, insulinoma - associated protein-2 (IA-2) and glutamic acid decarboxylase (GAD) [5,17,40]. GAD exists in two forms, GAD65 (65 KDa; 585 amino acids) and GAD67 (67 KDa; 593 amino acids). Only GAD 65 is expressed in the β-cells of human islets. GAD is a rate-limiting enzyme that catalyzes the conversion of glutamic acid to γ-amino butyric acid (GABA) [41]. Hsp 65 is a heat shock protein that is unique to mycobacteria [42]. It has been found that GAD65 and Hsp 65 have similar amino acid sequences [43]. In a study, it was found that up to 70 percent of T1DM patients have antibodies to GAD 65, compared to 4 percent of healthy individuals, thereby suggesting a cross reactive immune response targeted against GAD 65 [5]. This cross reactivity might pave the way for the destruction of the islet cells in genetically susceptible individuals [17]. More recent studies also support this hypothesis by proving that DNA vaccines involving mycobacterial HSP 65 protected NOD mice against diabetes [44]. Apart from this, a cause and effect experiment to serve as 'proof of principle' has never been reported to unequivocally support or rule out molecular mimicry as a cardinal process behind T1DM. The main reason for this could be non-availability of any practical and convenient model for *in vitro *or *in vivo *studies involving molecular mimicry.

It is not clear if molecular mimicry occurs also in cases of other chronic infections; individuals with T1DM are often susceptible to infection with chronic pathogens [45]. It has been shown that *Helicobacter pylori *(*H. pylori*), which causes gastritis, peptic ulcer and gastric cancer, is common in T1DM [46]. Certain studies found a higher prevalence of *H. pylori *infection in diabetic patients with a reduced glycemic control, while others did not support any correlation between metabolic control and *H. pylori *infection [47]. Interestingly it has been observed that the possibility of induction of Crohn's disease has also been reported after eradication of *H. pylori *[48,49].

## Prospective

Though MAP is identified as a trigger for T1DM, the underlying mechanism of MAP induced β-cell destruction is still an unsolved puzzle. As no easy animal model is available, development of an *in vitro *or an *in vivo *system will be of importance for deciphering the immunological network that is triggered in MAP pathogenesis, and that can be exploited for developing the functional evidence to link MAP to T1DM. It is also essential to understand the mechanisms by which mycobacteria such as MAP are acting as triggers in different autoimmune diseases and T1DM in particular. There is an urgent need to test a large number of geographically distinct patient populations with clinical T1DM in the same way as Sardinian patients have been tested for MAP. Also, it will be essential to develop high throughput genetic screens to identify single nucleotide polymorphisms that are associated with autoimmune diseases and those related to persistence of intracellular pathogens and a compensatory upregulation of proinflammatory cytokines. While it is important to identify other mycobacterial antigens that trigger host cell signaling cascades, it is certainly possible to scale up diagnostic screening tests based on existing antigens so that population level testing becomes practicable.

For decades, the population of Sardinia has been the subject of several human genetic analysis experiments. Population geneticists have revealed highly complex genetic relationships among Sardinians and other European populations. Peculiar gene pool characteristics of the Sardinians might have evolved over time as a function of strict isolation and consequent endogamy, consanguinity and the burden of historical scourges such as Malaria. High resolution, population based analyses are required to ascertain or negate any role of the unique Sardinian haplotypes and the high incidence of the autoimmune diseases.

Non-specific immune regulators such as *Mycobacterium indicus pranii *(MIP) [50] have been shown to play significant roles as immuno-therapeutic/prophylactic for leprosy, tuberculosis, leishmaniasis, psoriasis, bladder cancer and ano-genital warts [reviewed elsewhere - [51,52]]. It appears that non-specific immune stimulation could be the basic mechanism underlying the clearance of the lesions in all the above diseases when MIP was used. By analogy, MIP could also be used as a potential therapeutic/prophylactic agent for the autoimmune diseases rampant in Sardinia and beyond. It is essential therefore to test this organism in a setting such as Sardinia [53] as it has already worked wonderfully in a high burden country such as India which is crippled with tuberculosis, HIV, and diabetes. Given this, it will be tempting to propose experimental administration of MIP to susceptible populations and evaluate if it retards early onset of T1DM. Finally, the roles of intestinal microbiota and probiotic organisms should be explored at the interface of gut permeability and mucosal immunity [54] and the regulations thereof. Advancements in metagenomics of the gut microbiota and the consequent metabolomics and systems biology projects would be able to unravel the complex interactions underlying the alternative microbial mechanisms of T1DM apart from chronic MAP infection.

## Conflict of interests

The authors declare that they have no competing interests.

## Authors' contributions

PSR surveyed literature and developed text draft of the review. NA refined the write up, sketched figure-1 and wrote the prospective. LAS and NA supervised the Sardinian diabetes/MAP association studies that form the core concept of this article.

All authors have read and approved the final manuscript.
